# Sodium reduction and the correction of iodine intake in Belgium: Policy options

**DOI:** 10.1186/0778-7367-70-10

**Published:** 2012-05-30

**Authors:** Stefanie Vandevijvere

**Affiliations:** 1 Scientific Institute of Public Health, Department of Public Health and Surveillance, J.Wytsmanstraat 14, Brussels, 1050, Belgium

**Keywords:** Sodium, Iodine, Belgium

## Abstract

Many studies suggest that high salt intakes are related to high blood pressure and consequently cardiovascular diseases. In addition salt intake was found to be related with obesity, renal stones, osteoporosis and stomach cancer. Belgium, such as other European countries, is suffering from both salt intakes that are twice as high as the recommended intakes and mild iodine deficiency. No comprehensive strategy encompassing both public health problems has been developed. While specific salt reduction targets for processed foods are still under discussion using a consensus approach with industry, an agreement was signed between the bakery sector and the Ministry of Health in April 2009, to encourage and increase the use of iodised salt in the production of bread. Based on results of recent surveys on population iodine status it is advised not to currently revise iodine concentrations in salt in bread but to advocate for a higher percentage of bakers using iodised salt and to install a good monitoring system to control the percentage of bakers effectively using adequately iodised salt. With regard to salt reduction, it is of utmost importance that all companies contribute and harmonise the salt content of their products according to the lowest possible thresholds in a first step. In order to achieve this goal, it will be necessary, in addition to the consensus approach, to come up with at least some legislative tools such as a salt tax or mandatory labelling of foods exceeding a specific sodium concentration. Once salt reduction targets have been clearly defined in Belgium over the longer term, a legal framework should be set in place where iodine concentration in salt for the production of bread and household salt is strictly regulated by law, to avoid a large variability in the iodine content of salt brands consumed. In conclusion, it is possible to tackle salt reduction and iodine deficiency at the same time on the condition that the approach is coordinated and well monitored. All the interventions and measures taken should clearly include education and communication directed towards consumers, food producers, public health professionals, pharmacists, healthcare workers, and media representatives.

## Background

Although there is still controversy about the impact of sodium reduction on health or the benefits of universal salt reduction [1-3], it has been estimated on the basis of results of a meta-analysis of randomised controlled trials that a decrease of habitual salt intake with 6 g a day would result in reductions in systolic/diastolic blood pressure of 7/4 mmHg in people with hypertension and 4/2 mm Hg in those without hypertension. At the population level this would lead to an average lower rate of 24% for stroke and 18% for coronary heart disease [4]. A similar reduction in salt intake is also strived for in Belgium. Some authors suggest that a reduction of dietary salt of 3 g a day would have approximately the same effect on reducing cardiac events as a 50% reduction in tobacco use or a 5% reduction in body mass index among obese adults [5]. There is also evidence that for optimal health benefit salt intake should rather be reduced to 3 g per day [6] instead of less than 5 g per day as currently recommended by the World Health Organisation (WHO) [7]. In addition, increasing evidence suggests that salt intake is related to obesity through soft drink consumption and associated with renal stones, osteoporosis and stomach cancer [8]. Many studies have shown that salt reduction is highly cost-effective [5,9-11]. In the UK, close collaboration between government and industry on a voluntary basis, in reformulating many food items towards a lower salt concentration, introducing a successful traffic light labelling system and extensive awareness campaigns, have lead to a reduction of only 0.9 g of salt a day (10%) in population salt intake (from 9.5 to 8.6 g) in four years [12]. Finland initiated a successful approach to reduce salt intake already in 1970, *via* awareness media campaigns, collaboration with food industry and mandatory labelling of foods high in salt content. The average salt intake of the Finnish population was reduced from 14 g/day in 1972 to less than 9 g/day in 2002 [13-15]. These examples show that while salt reduction initiatives can be very successful, it is very difficult to reach the recommended salt intake level of less than 5 g per day at population level and it may take a tremendous amount of time to achieve this.

Belgium, such as other European countries, is also suffering from salt intakes [16,17] that are much higher than the recommended intake level of less than 5 g/day [7] or the level of 3 g/day recommended for optimal health benefit [6]. In addition it is important to note that Belgium since long has been suffering from mild iodine deficiency (MID) [18-21], which was found to represent a substantial economic burden to the Belgian health care system [22]. Both optimizing iodine intake and reducing sodium intake were set as priorities in the National Nutrition and Health Plan of the Belgian Ministry of Health 2005–2010 but no comprehensive strategy encompassing both public health problems has been developed until now.

## Discussion

While specific salt reduction targets for processed foods are still in the process of discussion using a consensus approach with industry, a selective and progressive salt iodisation program was initiated in Belgium. An agreement was signed between the bakery sector and the Ministry of Health in April 2009, to encourage and increase the use of iodised salt in the production of bread [23]. A national survey on iodine status in Belgian school-aged children initiated 17 months after the start of the fortification (autumn 2010) showed a median urinary iodine concentration (UIC) of 113.1 μg/L, compared with 84.8 μg/L among their mothers [24]. These results indicated iodine sufficiency among children and suggested that the voluntary salt in bread iodisation program may have contributed to the optimization of iodine intake in school-aged children, as in 1998 median UIC among school-aged children was only 80 μg/L [18]. In contrast, the median UIC during pregnancy, measured during the same time period (September 2010-June 2011) (124.1 μg/L) indicated MID in Belgium (S. Vandevijvere, unpublished observations). The percentage of households using iodised household salt in Belgium was only 37% [24].

The salt reduction initiative is managed by the Federal Public Service of Health in the framework of the National Nutrition and Health Plan. A working group has been set up consisting of the Federation of the food industry and all sub-sector federations, the Federation of the distribution sector, consumer organisations and scientific experts in the domain of food technology, food consumption surveys and iodine deficiency. Salt reduction targets are currently being defined and refined using a voluntary consensus approach with industry and gradually over time. It is however of utmost importance that all sectors and companies contribute to salt reduction so that similar products have similar salt content and the consumer can more easily adapt to the lower salt concentrations. Studies carried out by the World Action on Salt (WASH) [24] have proven that salt content in similar products may vary widely from brand to brand and salt content in identical products may even vary widely from country to country. This is proof of the fact that it could be so easy for the food industry to further reduce salt content of their foods. WASH is currently dealing with multinationals in order to try to control this issue. Also the European Commission should take further action within the High Level Group on Nutrition and Physical Activity and define clear reduction targets together with multinationals. They should be encouraged to harmonise the salt content of their products according to the lowest possible thresholds. However, in order to achieve this goal, it will be necessary, in addition to the consensus approach, to come up with at least some legislative tools such as a salt tax for the producers, a marketing tax for products high in salt or mandatory labelling of foods exceeding a specific sodium concentration, to make industry cut salt levels until technological and safety limits are reached.

The potential public health and economic benefits of a salt tax as part of a range of salt reduction interventions has been identified in modelling work [10]. In addition there has been a clear impact of existing taxes on protecting public health from tobacco-related harm [25] and alcohol consumption [26]. Mandatory labelling of foods exceeding a specific sodium concentration has been proven effective in Finland [15]. Only in those cases where legislative measures in combination with the consensus approach are not effective enough, salt concentrations in foods could be limited by law. An Australian study showed that the advantages for public health would be much greater when salt concentration in foods would be limited by law instead of using a consensus approach with industry [27]. However, this would decrease flexibility of the step-wise salt reduction program. Finally, although table salt is not the biggest contributor to salt intake in West European countries [28], legislative measures should be accompanied by mass awareness and educational campaigns to reduce the use of household salt by the caterers (in school canteens, hospitals and companies) and consumers, highlighting the fact that any used household salt should be iodised.

Concerns have been raised that programmes to reduce dietary salt could adversely impact programmes to prevent iodine deficiency disorders (and *vice versa*). However, iodine levels can be increased in salt to adjust for the recommended reduction in dietary salt to less than 5 g a day [29]. The most critical issue in optimizing both iodine and sodium intakes is to ensure coordination of both public health programmes and to advocate for adequate monitoring of both sodium and iodine intake among the population and sodium and iodine concentration in foods. In addition the impact of salt reduction measures on iodine intake should be calculated *via* scenario analyses, such as in the Netherlands where it was estimated that a reduction of salt intake with 50% would lead to a reduction of 10% in iodine intake among the population [30].

In a first phase one should focus purely on salt reduction. However it will be difficult to obtain the recommended level of less than 5 g/day. Therefore also new technologies such as inhomogeneous distribution of salt in products in order to obtain the same salt perception at lower concentrations, taste enhancers, and salt replacers will need to be considered [31-33]. This recommendation has also been published by the Belgian Superior Health Council (advice nr. 8663 of April 4, 2012).

In the case of Belgium, in the first instance, as long as the salt reduction targets are not yet completely defined and implemented, iodine nutrition policy should be further developed. The children’s survey showed a positive impact of the voluntary salt in bread iodisation program on the iodine status. Consequently it is important to continue optimising iodine status among children as 39% of the children were still found to be iodine deficient, and improving iodine status among women of child-bearing age and pregnant women, selectively and progressively in order to avoid intakes above the upper tolerable intake level and too rapid increases of intakes which may have adverse health effects [34]. Data from ESCOSALT showed that salt supply remained constant over the last 10 years in Belgium and % of use of iodised salt among the bakers increased from 11% (2001) to 44% (2011). Because Belgian children are currently iodine sufficient and the percentage of bakers actually using iodised salt in the production of bread is still quite low, it is advised not to currently revise concentrations of 10–15 mg/kg iodine in salt in bread but to advocate for a higher percentage of bakers using iodised salt in the near future *via* additional awareness campaigns and to install a good monitoring system by the Food Safety Agency to assess the percentage of bakers effectively using adequately iodised salt in the production of bread. In addition the use of iodised instead of non iodised household salt should be enabled in order to increase the proportion of the population using adequately iodized household salt, which is currently low in Belgium. However, this should not induce individuals to perceive that increased salt consumption is needed to prevent iodine deficiency. A certain increase in iodine intake among the population could also be obtained by highlighting the few food sources rich in iodine for the general public. Pregnant women should in addition be advised to take iodine-containing multivitamins starting before pregnancy or as soon as they become pregnant because the iodine status of women of child-bearing age in Belgium is not optimal and thus women enter pregnancy with a suboptimal iodine status.

Once salt reduction targets have been clearly defined in Belgium over the longer term, a legal framework should be set in place where iodine concentration in salt for the production of bread and household salt should be strictly regulated by law, to avoid a large variability in the iodine content of salt brands consumed. In addition its use in other food products should be discouraged. Prohibition however is difficult as in the past the Netherlands had to abandon their absolute ban on iodine fortification after decision by the European court of justice. It should also be possible to make timely adaptations considering results of monitoring surveys and taking into account reducing salt concentrations in products and decreasing sodium intake by the population. Sodium reduction targets should not be regulated by law but in case of lacking progress, mandatory labelling of foods too high in salt or a salt tax should be considered. Regulating salt concentrations by law should be the last option.

Sodium reduction in packaged and prepared foods other than bread is not expected to change the population’s iodine status. As bread is the main contributor to salt intake in Belgium [16] and it has been proven that further salt reduction in bread is possible without consumer aversion [35] and technological limits, the legislation of 2% salt in bread on dry matter basis needs to be adapted in future, which means that also iodine concentration in salt used for the production of bread will need to be increased and fixed by law. The optimal concentration needs to be calculated *via* scenario analyses [30]. In the past concentration of salt in bread was decreased already without notice of the consumer. At present assuming an iodine concentration in salt of 15 mg/kg, 2% salt in bread on dry matter basis, consuming 5 slices of bread contributes 30 μg of iodine to the daily intake. In addition, when use of household salt decreases over time due to salt reduction awareness campaigns and to avoid perception of individuals to higher salt intake in order to prevent iodine deficiency, it might be an option to consider iodisation of salt in other food items than only household salt and bread in order to ensure an optimal iodine status. This approach could also be particularly relevant for population groups who do consume less or no milk and dairy products, which are shown to be still the main contributor to iodine intake in Belgium [36]. In the Netherlands for example the potassium iodine content of table salt for bread making, which until 1996 had been 55 to 65 mg/kg was increased to 70 to 85 mg/kg, and the iodine content of table salt for domestic use was set at 30 to 40 mg/kg [37], because iodine supply was too low [38]. In 1999 the number of products to which iodised salt could be added was expanded in line with changing dietary habits to include breakfast cereals, breakfast biscuits, crisp bread and pickling brine in the production of meat products [39].The exploration of alternative vehicles to salt for iodine fortification could be investigated but seems less promising as salt is consumed by most of the population at fairly constant levels throughout the year and its taste and appearance is not affected by iodization. In addition salt iodization is highly cost-effective and recommended by the WHO [40].

## Conclusion

In conclusion, it is possible to tackle salt reduction and iodine deficiency at the same time on the condition that the approach is coordinated and well monitored. All the interventions and measures should clearly include education and communication directed towards consumers, food producers, public health professionals, pharmacists, healthcare workers, and media representatives. In addition reducing salt and optimizing iodine intake should never induce increase of intake of other unhealthy food substances such as sugar and fat.

## Competing interests

The author declares not having any conflict of interest
